# Comments on the Observational Study on Statin Intensity Following CABG

**DOI:** 10.1002/clc.70198

**Published:** 2025-08-28

**Authors:** Murat Abdulhamit Ercişli, Ahmet Süsenbük

**Affiliations:** ^1^ Department of Cardiovascular Surgery Gaziantep City Hospital Gaziantep Türkiye; ^2^ Department of Cardiology Abdülkadir Yüksel State Hospital Gaziantep Türkiye

Dear Editor,

We read with interest the article titled “Effect of Statin Intensity on Cardiovascular Outcomes and Survival Following Coronary Artery Bypass Grafting” recently published in Clinical Cardiology [1]. The study addresses a crucial area concerning optimal lipid management in patients undergoing Coronary Artery Bypass Grafting (CABG), particularly the impact of statin intensity on long‐term cardiovascular outcomes.

While we commend the authors for conducting this relevant and timely observational study, we would like to raise several points for clarification and discussion, which might significantly impact the interpretation of the results:

First, we note considerable discrepancies in patient numbers between the comparison groups: No‐statin (156 patients), low/moderate‐intensity statin (1301 patients), and high‐intensity statin (397 patients). Although the authors acknowledged this statistical concern due to the observational study design, such imbalanced group sizes may inherently introduce bias and confounding, limiting the reliability and generalizability of the conclusions.

Second, the authors mentioned that older patients and women were less likely to receive statins or were prescribed lower‐intensity statins. This finding raises concerns regarding potential selection bias or disparities in clinical practice. It would be helpful for the authors to elaborate further on possible reasons for these discrepancies and their potential influence on clinical outcomes.

Third, the study did not adequately track patient compliance or continued usage of statins over the follow‐up period, which is pivotal to understanding the true impact of the medication. Given that statin adherence significantly influences clinical outcomes, this limitation might have considerably affected the study's conclusions.

Additionally, the authors defined Major Adverse Cardiovascular Events (MACE) broadly to include acute coronary syndrome (ACS), cerebrovascular accident (CVA), and cardiovascular mortality. However, the study did not consider graft occlusion rates directly, which could significantly affect revascularization rates and subsequent MACE. Including graft occlusion data might have provided additional critical insights into statin efficacy.

Lastly, the timing of lipid measurements, which were taken variably between 1 and 3 months postoperatively, could introduce measurement bias. This variability in follow‐up LDL measurements might limit the robustness of the conclusions drawn about the efficacy of lipid management.

Despite these concerns, the findings strongly suggest potential long‐term benefits associated with high‐intensity statin therapy in reducing cardiovascular risks post‐CABG, especially evident beyond 2 years. This underscores the importance of robust randomized controlled trials to conclusively establish the most effective lipid‐lowering strategies in post‐CABG patients.

We appreciate the authors' efforts in highlighting this critical issue and look forward to further research addressing these areas of concern.
